# Susceptibility of tree shrew to SARS-CoV-2 infection

**DOI:** 10.1038/s41598-020-72563-w

**Published:** 2020-09-29

**Authors:** Yuan Zhao, Junbin Wang, Dexuan Kuang, Jingwen Xu, Mengli Yang, Chunxia Ma, Siwen Zhao, Jingmei Li, Haiting Long, Kaiyun Ding, Jiahong Gao, Jiansheng Liu, Haixuan Wang, Haiyan Li, Yun Yang, Wenhai Yu, Jing Yang, Yinqiu Zheng, Daoju Wu, Shuaiyao Lu, Hongqi Liu, Xiaozhong Peng

**Affiliations:** 1 National Kunming High-Level Biosafety Primate Research Center, Institute of Medical Biology, Chinese Academy of Medical Sciences and Peking Union Medical College, Yunnan, China; 2 State Key Laboratory of Medical Molecular Biology, Department of Molecular Biology and Biochemistry, Institute of Basic Medical Sciences, Medical Primate Research Center, Neuroscience Center, Chinese Academy of Medical Sciences, School of Basic Medicine Peking Union Medical College, Beijing, China

**Keywords:** Microbiology, SARS-CoV-2

## Abstract

Since severe acute respiratory syndrome coronavirus 2 (SARS-CoV-2) became a pandemic event in the world, it has not only caused huge economic losses, but also a serious threat to global public health. Many scientific questions about SARS-CoV-2 and Coronavirus disease (COVID-19) were raised and urgently need to be answered, including the susceptibility of animals to SARS-CoV-2 infection. Here we tested whether tree shrew, an emerging experimental animal domesticated from wild animal, is susceptible to SARS-CoV-2 infection. No clinical signs were observed in SARS-CoV-2 inoculated tree shrews during this experiment except the increasing body temperature particularly in female animals. Low levels of virus shedding and replication in tissues occurred in all three age groups. Notably, young tree shrews (6 months to 12 months) showed virus shedding at the earlier stage of infection than adult (2 years to 4 years) and old (5 years to 7 years) animals that had longer duration of virus shedding comparatively. Histopathological examine revealed that pulmonary abnormalities were the main changes but mild although slight lesions were also observed in other tissues. In summary, tree shrew is less susceptible to SARS-CoV-2 infection compared with the reported animal models and may not be a suitable animal for COVID-19 related researches. However, tree shrew may be a potential intermediate host of SARS-CoV-2 as an asymptomatic carrier.

## Introduction

The infectious disease caused by severe acute respiratory syndrome coronavirus 2 (SARS-CoV-2) infection is known as Coronavirus disease (COVID-19). Since the first case of SARS-CoV-2 infection was reported in December 2019, it has been epidemic in the world for more than eight months. It causes a pandemic with more than 20 million confirmed cases and nearly 740 thousand deaths in addition to huge economic losses to the world^1^. Nevertheless, it is widely considered to be controllable. However, there are still some critical aspects that need to be further investigated in COVID-19 patients, such as cytokine storm, immunopathogenic damages, tropism of SARS-CoV-2, and other sources of SARS-CoV-2 infection besides bat and pangolin that are regarded as the origin of SARS-CoV-2^2–4^. From these points of view, studies of animals become essentially important. In fact, several animal models of COVID-19 have been recently reported in murine^5^, hamster^6^, ferret^7^, and non-human primate^8–11^, which recapitulate COVID-19 from different aspects. In terms of susceptibility to SARS-CoV-2, in addition to these experimental animals, domestic animals and pets are also investigated^12^. Cats, as a popular pet, could be an important source of SARS-CoV-2 infection due to their close relationship with human beings.

The tree shrew, also known as *Tupaia belangeris*, is genetically demonstrated to be close to primates^13–15^. Therefore, it is being developed to be an experimental animal that could be an alternative to primates in biomedical research due to its unique characteristics^16^. In fact, tree shrew has been used for several animal models of virus infections, including hepatitis B^17^, influenza virus^18–20^, and Zika virus^21^. However, *Tupaia* model of high pathogenic viruses has not been reported yet, including SARS-CoV-2. Several reports show that SARS-CoV-2 may originate from wild animals^2–4^. Replication of SARS-CoV-2 in tree shrews is still unknown. In this study, in order to determine the possibility of tree shrew as a COVID-19 model, we tested the susceptibility of tree shrew to SARS-CoV-2 infection. We found that SARS-CoV-2 had limited replication and shedding in tree shrew, and cause mild histopathology, but no typical symptom is observed in infected tree shrew as reported in COVID-19 patients. The findings in the present study suggested that tree shrew may not be a suitable model of COVID-19 but a potential intermediate host of SARS-CoV-2.

## Results

### Clinical signs in SARS-CoV-2 infected tree shrew

**Figure 1:**
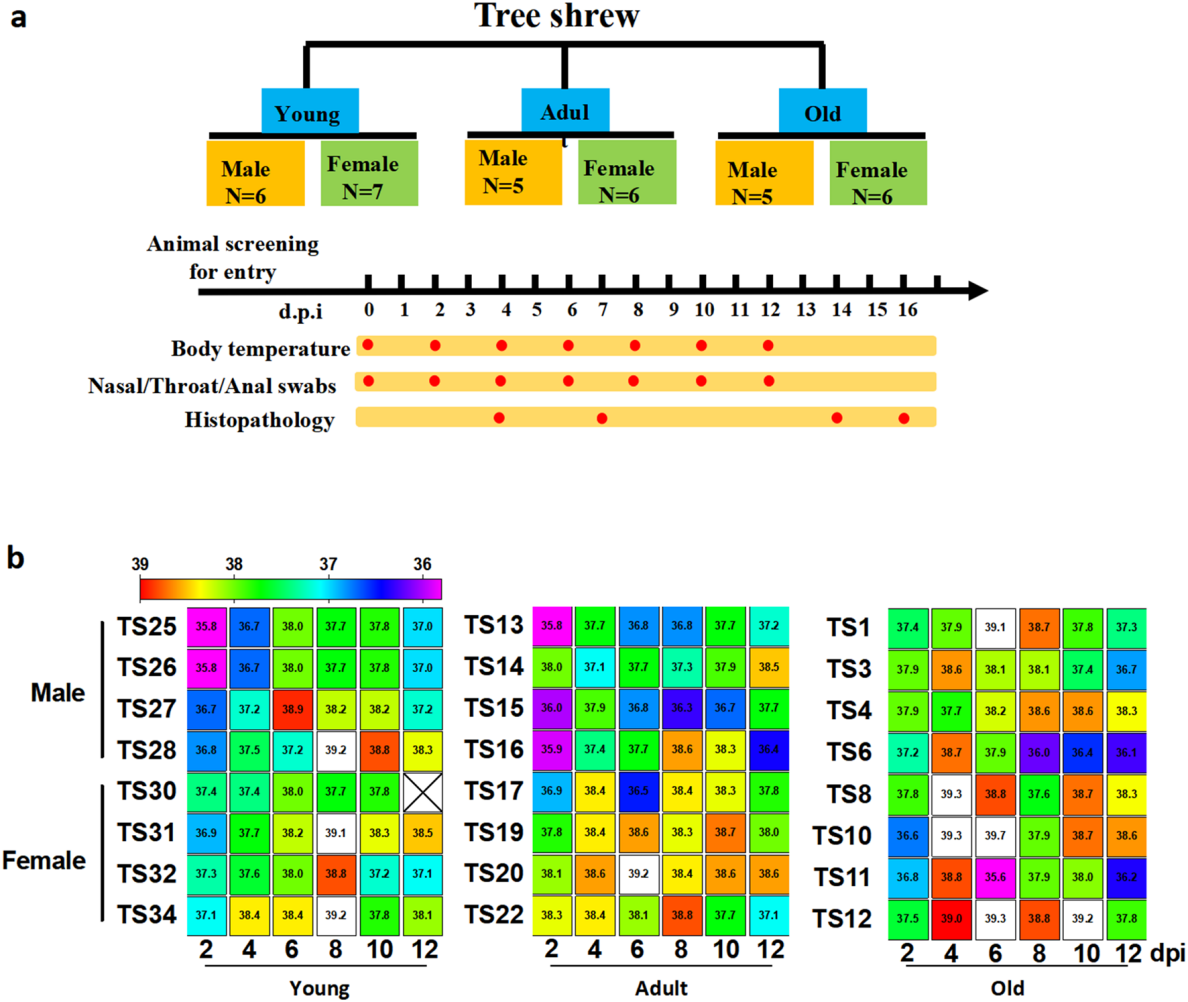
Study design and body temperature in SARS-CoV-2 infected tree shrews. (**a**) Total 35 tree shrews (*Tupaia belangeris*) were used for infection in this study. Two challenge experiments were conducted with 1 ml 10^6^ pfu SARS-CoV-2 nasally (500 μl/each). Twenty-four animals for the first experiment were divided into three groups (young, adult and old) according to ages. Each group included half male and half female. Following the viral inoculation, clinical observation and viral load assay were performed as indicated. Animals were dissected in about two weeks post viral inoculation. (**b**) On every other day as indicated in (**a**), body temperature of tree shrew was monitored and recorded. The software Graphpad was utilized for data processing and plotting as rainbow heat map. X represents no data collected. Body temperature beyond 39 °C was shown in white boxes.

During the period of this study, we couldn’t observe any other clinical sign besides change of body temperature. After SARS-COV-2 inoculation, body temperature of tree shrew was monitored every other day. Among three ages of virus-inoculated tree shrews, all young tree shrews except one showed the increased body temperature. There are 5 young tree shrews (2 males, 3 females) with the peak body temperature on 6 or 8-day post inoculation (dpi), followed by a gradual decline. The peaks of body temperatures in two females and one male young tree shrews were above 39 °C (Fig. 1a). In addition, among old tree shrews, one male and three female had the peak body temperature (> 39 °C) on 4 or 6 dpi. However, only one adult female showed the peak body temperature (39.2 °C) on 6 dpi (Fig. 1b). These results indicated that young/old tree shrews and female tree shrews showed higher sensitivity to SARS-CoV-2 infection, as compared to adult and male tree shrews.

### Virus shedding from SARS-CoV-2 infected tree shrew

Genomic RNA of SARS-CoV-2 became detectable on 6 dpi in 5 nasal swabs, 3 throat swabs, 2 anal swabs and one serum sample from young tree shrews, in 2 nasal swabs from adult tree shrews and in 2 nasal swabs, 1 anal swab from old tree shrew (Table 1). Notably, four samples (nasal, throat, anal swabs and blood) collected from one young tree shrew all showed virus RNA positive. The highest copy number of viral genomic RNA was 10^5.92^/ml in nasal swab from the young tree shrew. On 8 dpi, there were four young, three adult and four old tree shrew with viral RNA positive in some of samples. The highest level of virus shedding was still observed in the young tree shrew. On 12 dpi, the young tree shrews showed the decreased virus shedding and only the animal TS27 had RNA-positive throat swab. In contrast, increased number of old tree shrews had detectable viral RNA in nasal, throat, and anal swabs (Table 1). From these findings, we deduced that young tree shrew was more susceptible to SARS-CoV-2 infection than adult and old tree shrew at the early stage of SARS-CoV-2 infection. However, old and adult tree shrews gave longer duration of virus shedding than young tree shrews. Moreover, we noticed that more male animals of adult and old tree shrews showed virus shedding than females.

**Table 1:**
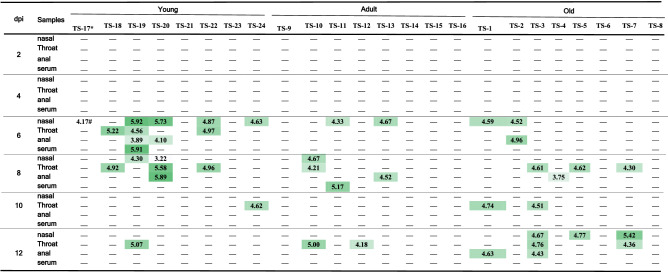
Copy number of viral genomic RNA in clinical samples from SARS-CoV-2 infected tree shrews.

Density of green color is proportional to the copy number of viral genomic RNA.

*Animal code.

^#^Copy number of viral genomic RNA was expressed as log_10_/ml.

−Means undetectable.

### Viral load in tissue samples from SARS-CoV-2 infected tree shrew

In order to determine viral load in tissue samples, we dissected 19 animals from 3 ages of tree shrews on 4, 7 dpi and 14/16 dpi (Table 2). Sixteen major tissues were collected from each animal. Viral genomic RNA was quantified as described in “Methods”. On 4 dpi, the early stage of SARS-CoV-2 infection, we could detect viral RNA from 1–2 tissues in all three ages of tree shrews. In the young tree shrews, we detected viral RNA from seven tissues on 7 dpi. However, only the lung tissues showed viral RNA positive on 14 dpi. These results suggest that SARS-CoV-2 causes the early infection in young tree shrews, which is consistent with the virus shedding. The viral loads in the tissues from the old tree shrews were similar to those in the young tree shrews. In contrast, in the adult tree shrews, viral RNA was detected in four tissues on 7 dpi and in seven tissues on 14 dpi, including the spinal cord and uterus. During SARS-CoV-2 infection, we detected viral RNA in the lung tissues from more than half dissected animals (10/19), suggesting lung is the main site for viral replication, followed by the digestive tissues (4/19) and heart (4/19). The highest viral load (10^9.08^/ml) was detected in the pancreas from one adult tree shrew dissected on 14 dpi. TCID50 results suggested that viral RNA in tissues could be infectious although the titer was low (0–3.5 log10/ml) (Table S1). We further determined the mRNA expression of ACE2, the SARS-CoV-2 receptor, in various tissues from the uninfected young, adult and old tree shrews. We found that the young tree shrews had relatively high mRNA expression of ACE2, especially in duodenum, pancreas, colon, stomach, penis, and heart. The levels of ACE2 mRNA in the old tree shrews were secondary to those in the young tree shrews. Very low levels of ACE2 were found in the tissues from the adult tree shrews (Table S2). These results indicated that SARS-CoV-2 could replicate in multiple tissues of tree shrews to a certain extend and the ACE2-independent mechanism underlying SARS-CoV-2 infection may exist in tree shrews.

**Table 2:**
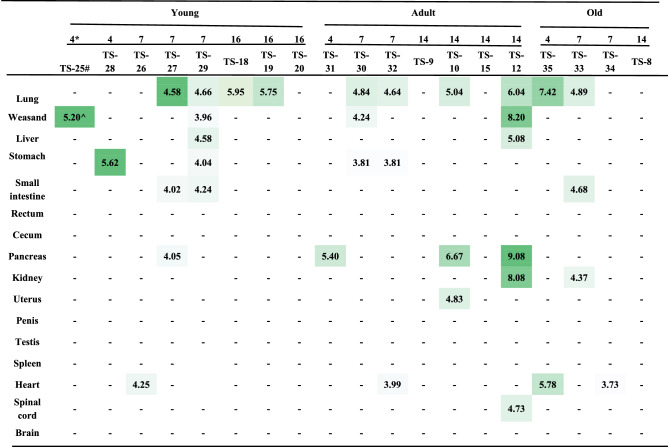
Viral load in tissues collected from SARS-CoV-2 infected tree shrews.

Density of green color is proportional to the copy number of viral genomic RNA.

*Days post virus inoculation.

^#^Animal code.

^Copy number of viral genomic RNA was expressed as log_10_/ml.

−Means undetectable.

### Histopathological characteristics in SARS-CoV-2 infected tree shrew

**Figure 2:**
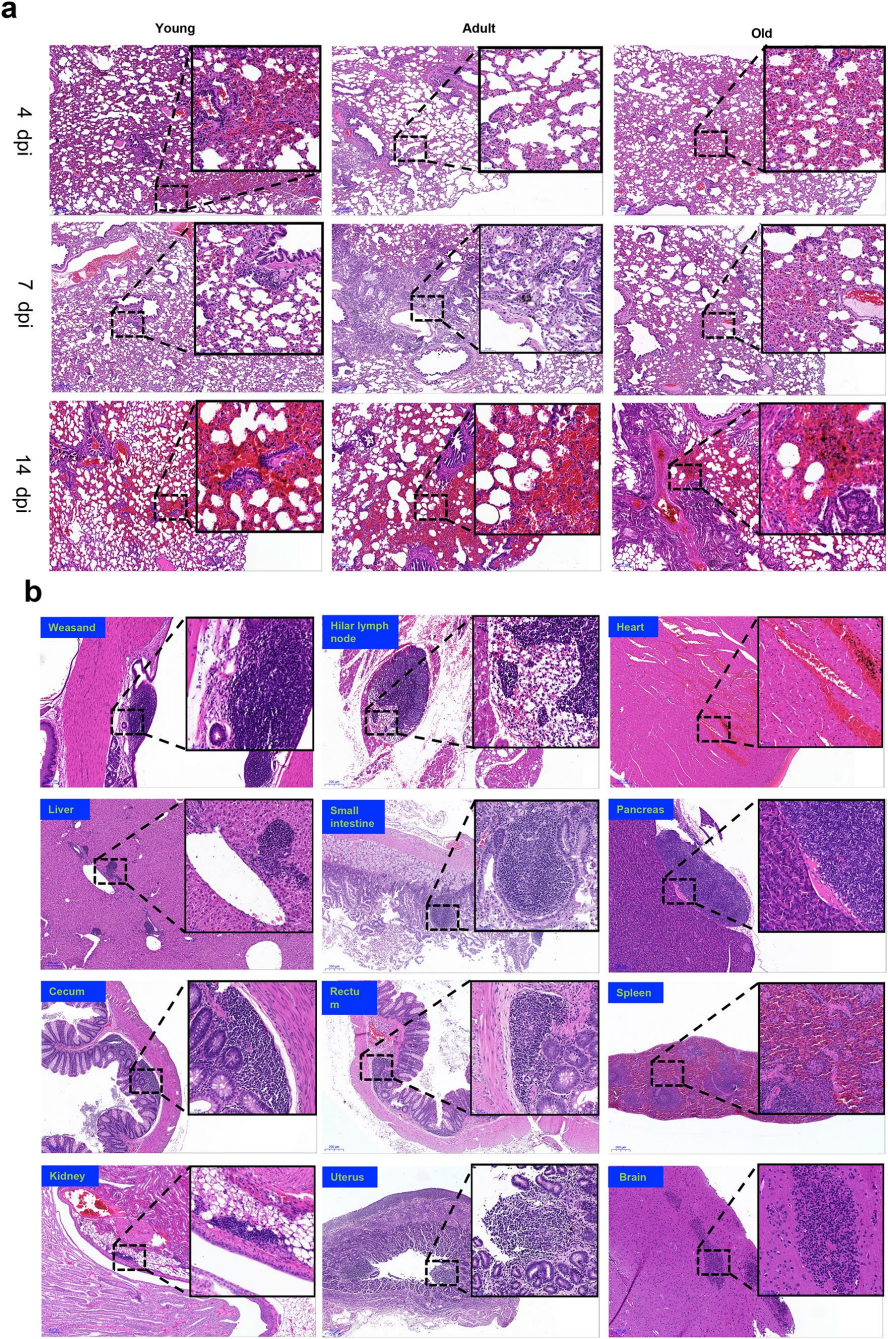
Histopathological examination in SARS-CoV-2 infected tree shrews. On 4, 7 and 14/16 dpi, tree shrews were euthanized and dissected. Tissues were collected from each animal for H&E staining and histopathological analysis. The histological sites with typical and representative lesions were zoomed in, which was described in text. (**a**) Histological lesions in the pulmonary tissues. The representative images were shown here from young, adult and old tree shrews dissected at the different stages of SARS-CoV-2 infection. (**b**) Histopathological changes in the other twelve tissues. Each image here represents one tissue of an animal, including weasand, Hilar, heart, liver, small intestine, pancreas, cecum, rectum, spleen, kidney, uterus, brain. Histopathological score of tissues in all tree shrews was summarized in Table 3.

To determine the host response to SARS-CoV-2 infection, we examined sixteen tissues from each of all 35 tree shrews dissected. No gross lesion was observed in any organs of infected tree shrews. Histopathological inspection revealed that 13 out of 16 tissues had various degrees of pathological changes (Table 3). The lungs of tree shrews in all age groups showed widened pulmonary septum, hyperemia of interstitium, airway obstruction, consolidation of lung margin, local hemorrhagic necrosis, and infiltration of inflammatory cells. Inflammatory cells infiltrated into the submucosa of the trachea in the aged group (Fig. 2a). In the other non-pulmonary tissues, we found lymphatic nodules in weasand, pancreas, small intestine, rectum and uterus, the congestion in heart and spleen, the hyperplasia of intestinal gland in the small intestine, the inflammatory cell infiltration in portal area of liver, renal pelvis, cecum, microglia in the basal part of the brain (Fig. 2b). Overall evaluation of histopathology in tissues from all 35 tree shrews revealed that all tissues, except lung, showed only mild histological abnormality. Lung was the major organ affected by SARS-CoV-2 infection. Mild (+) pulmonary abnormality was observed in 60.0%, intermediate (++) abnormality in 17.1% and severe (+++) in 8.6% of 35 tree shrews. Unexpectedly, we also found some mild histopathological changes in brain, heart, liver and pancreas (Table 3).

**Table 3:** Summary of histopathological examination of tissues from all 35 tree shrews.

	Young				Adult				Old			
Severity	−	+	++	+++	−	+	++	+++	−	+	++	+++
Lung	2/13*	**7/13**	**3/13**	**1/13**	**2/11**	**6/11**	**2/11**	**1/11**	**1/11**	**8/11**	**1/11**	**1/11**
Weasand	**12/13**	**1/13**	**///**	**///**	**8/11**	**3/11**	**///**	**///**	**10/11**	**1/11**	**///**	**///**
Liver	**6/13**	**7/13**	**1/13**	**///**	**4/11**	**6/11**	**1/11**	**///**	**4/11**	**6/11**	**1/11**	**///**
Small intestine	**9/13**	**3/13**	**1/13**	**///**	**9/11**	**2/11**	**///**	**///**	**8/11**	**3/11**	**///**	**///**
Rectum	**10/13**	**3/13**	**///**	**///**	**9/11**	**2/11**	**///**	**///**	**7/11**	**4/11**	**///**	**///**
Cecum	**9/13**	**4/13**	**///**	**///**	**10/11**	**1/11**	**///**	**///**	**10/11**	**1/11**	**///**	**///**
Pancreas	**12/13**	**1/13**	**///**	**///**	**11/11**	**///**	**///**	**///**	**10/11**	**1/11**	**///**	**///**
Hilar lymph node	**13/13**	**///**	**///**	**///**	**10/11**	**1/11**	**///**	**///**	**10/11**	**1/11**	**///**	**///**
Kidney	**6/13**	**7/13**	**///**	**///**	**8/11**	**3/11**	**///**	**///**	**5/11**	**6/11**	**///**	**///**
Uterus	**6/7**	**1/7**	**///**	**///**	**4/6**	**2/6**	**///**	**///**	**4/6**	**2/6**	**///**	**///**
Spleen	**9/13**	**4/13**	**///**	**///**	**8/11**	**3/11**	**///**	**///**	**9/11**	**2/11**	**///**	**///**
Heart	**12/13**	**1/13**	**///**	**///**	**9/11**	**2/11**	**///**	**///**	**10/11**	**1/11**	**///**	**///**
Brain	**13/13**	**///**	**///**	**///**	**9/11**	**2/11**	**///**	**///**	**9/11**	**2/11**	**///**	**///**

*The ratio of animal number with pathological changes over the all dissected animals.

/// indicates no data.

## Discussion

The announcement of the genome of SARS-CoV-2 reveals the unique relationship of SARS-CoV-2 with 2003 SARS-CoV and 2012 MERS-CoV^22^, which not only attracts great attentions but also leads to many conjectures. The main concerns are the origin of SARS-CoV-2, the natural reservoir, intermediate host, factors affecting infection and prognosis, and so on. These are actually about the sensitivity or susceptibility of host to SARS-CoV-2 infection^12^. Although the retrospective studies of COVID-19 have given us some answers^23^, further investigation needs to be performed in various animals. So far, several animal models of COVID-19 have been reported to successfully recapitulate some aspects of this disease^6–9,24–26^ and also indicate that these experimental animals are susceptible to SARS-CoV-2 infection.

*Rhinolophus sinicus* is mostly recognized as the natural host for SARS-CoV-2^27^. However, the intermediate host is still controversial. Nevertheless, some domestic animals and pets, including cats, can be infected by SARS-CoV-2^12^. Our previous study revealed the variable susceptibility among three species of non-human primates to SARS-CoV-2 infection^8^. Here we experimentally infected tree shrew, an emerging experimental animal genetically close to NHP^14^. No typical clinical sign except the increasing body temperature was observed in SARS-CoV-2 infected tree shrews, although viruses were detectable in swabs, serum samples and some of tissues. These results indicate that tree shrew may be a potential intermediate host for SARS-CoV-2.

It is clinically reported that old COVID-19 patients show high morbidity and mortality, which is thought to be associated with the comorbidities^28^. Children confirmed as SARS-CoV-2 infection showed milder clinical symptoms than adult COVID-19 patients^29^. However, COVID-19 children are reported to be critical in fecal–oral transmission due to persistence of fecal virus shedding even though nasopharyngeal swabs are tested virus-negative^30^. Results in this study indicate that age may affect SARS-CoV-2 infection of tree shrew. Firstly, more young and old tree shrews showed increasing body temperature than adult tree shrews (Fig. 1b). Secondly, young tree shrews had more severe virus shedding at the early stage of virus infection than the other animals, and the old tree shrews had a longer duration of virus shedding than others (Table 1). One of young male tree shrew didn’t have significant increased body temperature, but virus shedding from nasal, throat, anal and serum was detected on 6 dpi. These results indicated the asymptomatic infection of SARS-CoV-2 in the young tree shrews. Clinical retrospective studies indicate that SARS-CoV-2 tends to affect men severely than women, which refers to the mortality*.* In other words, higher mortality rate is caused in men than women COVID-19 patients^31–34^. There are several factors may underlie this sex difference, including activity of the immune system and its modulation by sex hormones, coagulation pattern, and preexisting cardiovascular diseases as well as smoking and drinking habits^32^. In our study, in terms of viral loads in tissues and swab samples not mortality, we found that female tree shrews showed higher sensitivity to SARS-CoV-2 infection, as compared to male tree shrews. We didn’t observe the sex difference in the other aspects of SARS-CoV-2 infection.

Although SARS-CoV-2 infection didn’t cause severe disease in all three ages of tree shrews, viral replication and mild histopathological changes were still observed in this study. Particularly, we found the severe lung abnormality of histology in one adult tree shrew, which may be caused by immunopathological responses, such as cytokine storm^35^. Unfortunately, reagents for analysis of cytokines in tree shrews are very limited so that we couldn’t further evaluate levels of inflammatory cytokines although we may analyze mRNA levels of cytokines via RT-qPCR in the future.

Angiotensin-converting enzyme 2 (ACE2) has been demonstrated as an essential receptor for SARS-CoV-2 infection in vertebrate animals^36^. The interaction between ACE2 and RBD domain of viral spike protein determines the susceptibility and range of host to SARS-CoV-2 infection^37^. Furthermore, distribution of ACE2 in vivo may be associated with viral pathogenesis^38^. However, no profile of ACE2 in various tissues of animal models has been reported so far. Amino acid alignment of critical domain of ACE2 binding spike protein RBD showed that there were 10 amino acid different between tree shrew and human, whereas no difference exists between rhesus monkey and human^8^. This may be one of the reasons that tree shrews are less susceptible to SARS-CoV-2 infection compared with the reported animal models.

Tissue distribution of SARS-CoV-2 in tree shrew is different from that reported in autopsied COVID-19 patients. For example, viral genomic RNA was unexpectedly detected in kidney, pancreas, and spinal cord in SARS-CoV-2 infected tree shrew (Table 2). In particular, the highest viral load was observed in the pancreas of one adult animal among all analyzed tissues, which is not correlated with the levels of ACE2 mRNA (Table S2), suggesting the presence of other mechanisms underlying SARS-CoV-2 infection in tree shrews. Pancreatic injury has been reported in COVID-19 patients, possibly linking with diabetes^39^. However, the reported animal models of COVID-19, including NHP^8,10,11,40,41^, hamster^6,42,43^, ferret^7^, adapted Balb/c mice^44,45^, and transgenic mice^5,46–48^ hadn’t shown the presence of viruses and injury in pancreas. Interestingly, we observed in this study the viral loads and the mild histopathological changes in the pancreas tissues from some of tree shrews, suggesting that tree shrew may be used to study effects of SARS-CoV-2 on pancreas and the related diabetes in the future.

In conclusion, tree shrew is not as susceptible to SARS-CoV-2 infection as the reported animal models of COVID-19, though limited replication of SARS-CoV-2 and mild histopathology was observed in some tissues. In addition, commercial reagents and completely domesticated tree shrews are very limited. Therefore, tree shrew is not a suitable experimental animal for COVID-19 related studies. However, it should be very important to further investigate whether wild tree shrews in nature are infected by or asymptomatic carrier of SARS-CoV-2.

## Methods

### Experimental animals and ethics

Tree shrew, *Tupaia belangeri chinensis*, were bred in the non-barrier room of the Center of Tree Shrew Germplasm Resource (Use license: SYXK K2018-0002; Manufacturing license: SCXKK2018-0002), the Institute of Medical Biology, Chinese Academy of Medical Sciences. After quarantine and before infection, animals were transferred to ABSL-3 facility and housed in the isolated ventilation cages (one animal/cage). All animal procedures were approved by the Institutional Animal Care and Use Committee of Institute of Medical Biology, Chinese Academy of Medical Science (Ethics number: DWSP202002 001), and performed in accordance with relevant guideline and regulations in the ABSL-3 facility of National Kunming High-level Biosafety Primate Research Center, Yunnan China.

### Study procedures

Procedures in this study are outlined in Fig. 1a. Total 38 tree shrews were used in this study, 35 for two challenge experiments and 3 for analysis of ACE2 mRNA expression. Twenty-four animals for the first experiment were divided into three groups with consideration of gender and age, including 8 young (6 months to 12 months), 8 adults (2 years to 4 years) and 8 old (5 years to 7 years) groups. Each group contains half male and half female animals. After baseline data and samples were collected right before virus inoculation, each of tree shrew was inoculated with 1 ml of 10^6^ pfu SARS-CoV-2 nasally (500 μl/each nostril). Observation and analysis were performed as indicated in Fig. 1a . Necropsy was conducted in about 2 weeks post viral inoculation, followed by analysis of viral loads and histopathology. Additional eleven tree shrews (5 young, 3 adult and 3 old tree shrews) were used for the secondary infection experiment. After each of tree shrew was inoculated with 1 ml of 10^6^ pfu SARS-CoV-2 nasally (500 ul/each nostril), animals were dissected on 4 dpi or 7 dpi. The tissue samples were harvested for analysis of viral loads and histopathology.

### Quantification of viral genomic RNA

Swab samples soaked in Trizol solution were vortexed and swabs were removed. 200 μl of each sample were used for RNA extraction and purification via the kit Direct-zol RNA MiniPrep. Tissue samples were homogenized in Trizol and 200 μl of each for total RNA preparation. Briefly, 50 μl RNase/DNase-free H2O was used to elute RNA from the column. 7.5 μl of each RNA was analyzed in each well of 384-well plate by one-step RT-qPCR using gene-specific primers and probe as described before^8^.

### ACE2 mRNA expression in various tissues of tree shrew

Tissue samples were homogenized in Trizol and 200 μl of each for total RNA preparation. Briefly, 50 μl RNase/DNase-free H2O was used to elute RNA from the column. 7.5 μl of each RNA was analyzed by one-step RT-qPCR using gene-specific primers and probe as follows, Forward: ACACTTGCACCTCCTTACCA; Reverse: ACAAGCAGGATGACAATGCC;

Probe: 5 ‘Fam–ACCACTACCAGTCCCATCACAACTCCA-3′Tamra. The mRNA levels of ACE2 were normalized to that in the lung.

### TCID50 assay

The assay was performed in 96-well plate with 4 × 10^4^ cells/well. Cells were washed with PBS three times before addition of samples. The supernatant of tissue homogenate was serially diluted in DMEM without antibiotics and serum. 100 μl of diluted supernatant was added to each well that had been washed with PBS, followed by one-hour incubation at 37 °C. 100 μl of maintaining medium (1% antibiotics and 4% serum) was added to each well. Cells were continuously cultured for 96 h. Cytopathogenic effects were recorded for the calculation of TCID50 via Reed-Muench method.

### Histopathological analysis

Tissue samples of heart, liver, spleen, lung, kidney, weasand, stomach, small intestine, rectum, pancreas, brain, spinal cord, uterus, penis, testis, cecum were harvested and fixed in 10% neutral buffered formalin. Paraffin-embedded tissues were cut into 5 μm of sections, followed by hematoxylin and eosin (H&E) staining. Slides were scanned with 3DHISTECH and inspected by the experienced pathologist using the manufacture provided software CaseViewer.

## Supplementary information

Supplementary information.
