# Supplementary material for: Susceptibility of tree shrew to SARS-CoV-2 infection
Source: Sci Rep. 2020 Sep 29;10:16007. doi: 10.1038/s41598-020-72563-w (PMC7525503; doi:10.1038/s41598-020-72563-w)
Supplement: Supplementary file 1 — Supplementary information. [file 41598_2020_72563_MOESM1_ESM.docx]

**Supplementary Information**

**Susceptibility of tree shrew to SARS-CoV-2 infection**

Yuan Zhao^1#^, Junbin Wang^1#^, Dexuan Kuang^1#^, Jingwen Xu^1^, Mengli Yang^1^, Chunxia Ma^1^, Siwen Zhao^1^, Jingmei Li^1^, Haiting Long^1^, Kaiyun Ding^1^, Jiahong Gao^1^, Jiansheng Liu^1^, Haixuan Wang^1^, Haiyan Li^1^, Yun Yang^1^, Wenhai Yu^1^, Jing Yang^1^, Yinqiu Zheng^1^, Daoju Wu^1^, Shuaiyao Lu^1,2^*, Hongqi Liu^1^*, Xiaozhong Peng^1, 2^*

**Affiliations**

1. National Kunming High-level Biosafety Primate Research Center, Institute of Medical Biology, Chinese Academy of Medical Sciences and Peking Union Medical College, Yunnan China
2. State Key Laboratory of Medical Molecular Biology, Department of Molecular Biology and Biochemistry, Institute of Basic Medical Sciences, Medical Primate Research Center, Neuroscience Center, Chinese Academy of Medical Sciences, School of Basic Medicine Peking Union Medical College, Beijing China

# Equal contributions.

**Short Title:** SARS-CoV-2 infection of tree shrew

**Conflicts of interest:** The authors declare no conflicts of interest.

***Correspondence to:** Xiaozhong Peng (pengxiaozhong@pumc.edu.cn);

Hongqi Liu (lhq@Imbcams.com.cn);

Shuaiyao Lu (lushuaiyao-km@163.com)

Table S1

SARS-CoV-2 TCID50 in tissue samples from the infected tree shrews

Table S2

mRNA levels of ACE2 in uninfected tree shrews

**Table S1 SARS-CoV-2 TCID50 in tissue samples from the infected tree shrews**

| Animal# | Tissue | lgTCID_50_/ml |
| --- | --- | --- |
| TS25 | Weasand | 3.5* |
| TS28 | Stomach | -# |
| TS29 | Lung | 3.5 |
| TS30 | Lung | 2.5 |
| TS30 | Weasand | - |
| TS31 | Pancreas | 3.5 |
| TS35 | Heart | - |
| TS35 | Lung | - |

*Cytopathogenic effects were recorded for the calculation of TCID50

via Reed-Muench method.

# No cytopathogenic effect observed.

**Table S2 mRNA levels of ACE2 in uninfected tree shrews**

|  | **Young** | **Adult** | **Old** |
| --- | --- | --- | --- |
| Lung | 1.00 | 1.00 | 1.00 |
| Trachea | 0.06* | 0.25 | 13.90 |
| Bronchus | 0.22 | 0.37 | 0.00 |
| Pharynx | 0.00 | 0.07 | 0.05 |
| Esophagus | 0.04 | 0.01 | 0.17 |
| Liver | 0.06 | 0.06 | 0.43 |
| Stomach | 55.07 | 0.07 | /// |
| Duodenum | 475.68 | /// | 172.82 |
| Colon | 83.49 | 1.04 | 0.33 |
| Pancreas | 979.03 | 0.00 | 11.55 |
| Rectum | 8.40 | 3.85 | 11.55 |
| Muscle | 8.40 | 1.04 | 0.62 |
| Kidney | 8.40 | 0.05 | 11.55 |
| Bladder | 0.00 | 0.04 | 11.55 |
| Spleen | 1.04 | 0.52 | 1.34 |
| Heart | 11.44 | 0.00 | 11.55 |
| Eyeball | 0.03 | 0.00 | 0.05 |
| Spinal cord | 0.31 | 0.01 | 0.05 |
| Penis | 360.09 | 0.10 | 0.11 |
| Testis | 0.15 | 0.31 | 0.00 |

*The mRNA levels of ACE2 were normalized to that in the lung.
